# Postoperative analgesic effect of acupotomy combined with patient-controlled analgesia in patients undergoing video-assisted thoracoscopic surgery: a study protocol for a randomized controlled trial

**DOI:** 10.1186/s13063-020-04926-7

**Published:** 2020-12-04

**Authors:** Cai Jiang, Yinyan Li, Xiaomei Li, Jinhua Guo, Miaomiao Guo, Shengxian Yu, Zhonghua Lin

**Affiliations:** 1 Fujian Provincial Hospital, Fuzhou, Fujian China; 2 Outpatient Department of Guoyitang affiliated to Fujian University of Traditional Chinese Medicine, Fuzhou, Fujian China

**Keywords:** Acupotomy, Patient-controlled analgesia, Video-assisted thoracoscopic surgery, Randomized controlled trial, Protocol

## Abstract

**Background:**

Postoperative acute pain is a common issue following thoracic surgery. Acupotomy is a common and safe intervention method for pain treatment in clinical practice. In previous preliminary experiments, we found that acupotomy has a good clinical effect and good safety in the treatment of pain after thoracoscopic surgery. However, due to a lack of a rigorous design and an adequate sample size, its efficacy still requires further confirmation. The purpose of this study will be to explore the efficacy and safety of acupotomy combined with patient-controlled analgesia (PCA) for the treatment of pain after video-assisted thoracic surgery (VATS).

**Methods:**

The study will be a single-centre, parallel group, randomized controlled trial.

Seventy patients with significant pain after thoracoscopic surgery with a visual analogue scale (VAS) score ≥ 7 will be included and randomly distributed into two groups: G1, the acupotomy combined with PCA group; and G2, the conventional PCA group. The primary outcome measure is pain scores at rest and coughing evaluated with the VAS by a blinded observer in the postanaesthesia care unit (PACU) and postoperatively at 1, 2, 4, 8, 12, 24, 48, and 72 h. The secondary outcome measures are postoperative requirements for rescue analgesia, the cumulative amount of self-administered analgesics, the level of sedation (LOS), the Bruggemann comfort scale (BCS), and the functional activity score (FAS) concerning adverse effects and patient satisfaction.

**Discussion:**

This trial has the potential to identify an innovative and effective analgesic method for postoperative pain management for VATS. The findings may advocate for the inclusion of the treatment of comorbid pain after thoracoscopy in current pain management practice guidelines.

**Trial registration:**

Chinese Clinical Trial Registry ChiCTR1900027191. Registered on 4 November 2019

## Background

Postoperative acute pain is a common issue following thoracic surgery [1]. Although minimally invasive compared to the thoracotomy approach, postoperative pain arising from intercostal muscle, fascia, nerve, and visceral tissue injury after video-assisted thoracoscopic lung surgery must still be considered moderate to severe [2, 3]. Poor postoperative analgesia is not only related to reduced patient satisfaction but also leads to impairment of postoperative cardio-pulmonary function and further deterioration of the condition [4, 5]. For example, severe postoperative pain will increase the risk of long-term chronic pain, sympathetic activation may lead to cardiovascular adverse events (AEs), slow activity will increase the risk of thromboembolism events, and if the patient does not recover for a long time, he or she may have anxiety, depression, and other psychological problems [6]. The medical costs and hazards of postoperative pain, including health care costs and medical resource utilization, daily activity restrictions, reduced quality of life, and an increased risk for mortality, inflict a considerable burden on patients, families, and health care systems.

Patient-controlled analgesia (PCA) is a personalized strategy involving various opioids that allows patients to administer analgesics as needed and has been demonstrated to be effective for postoperative pain management under diverse conditions [7, 8]. In addition, a series of reports have shown that other effective analgesic techniques exist, including intraspinal block, peripheral nerve block, wound infiltration, and systemic nonsteroidal anti-inflammatory drug (NSAID) administration [9–11].

However, most of these modalities are technically complex and present a high risk and/or carry a high risk of serious side effects such as epidural haematomas, dural puncture, nerve injuries, pneumothorax, hypotension, infection, respiratory depression, cough suppression, coagulopathy, local anaesthetic toxicity, and renal impairment [12, 13]. Therefore, no analgesic drug can be used alone to effectively treat severe pain without side effects. Once again, multimodality pain management is found to be superior. Enhanced Recovery After Surgery (ERAS) is a multimodal, multidisciplinary approach to the treatment of surgical patients with the aim of enhancing the quality of recovery after surgery [14]. The role of pain management in ERAS pathways is fundamental considering the importance of containing surgical stress, reducing pain-related complications, and accelerating recovery [15–17]. In regard to pain management, ERAS promotes the adoption of a multimodal strategy that is tailored to individual patients [18]. However, no consensus is currently available on the best strategy for treating pain after video-assisted thoracic surgery (VATS).

Acupotomy, which is widely used in Korea and China as a special form of acupuncture with characteristics of surgical procedures, is widely used for musculoskeletal conditions and is considered an excellent treatment for pain relief [19]. The analgesic effect of acupotomy has been recognized by many clinicians [20]. Acupotomy can relieve muscle spasms, relax compressed nerves, promote local microcirculation, and thus alleviate ischaemia-related pain. An animal study also showed that extracellular signal-regulated kinase activation in the skin layer contributes to the analgesic effect of acupuncture [21]. Moreover, in previous preliminary experiments, we found that acupotomy has a good clinical effect and good safety in the treatment of pain after thoracoscopic surgery. The preliminary study did not set strict inclusion and exclusion criteria, had no randomized control, and included simple outcome indicators. However, due to the lack of a rigorous design and an adequate sample size, the efficacy requires further confirmation.

Thus, in this trial, we combined acupotomy with PCA to form a multimodal analgesia regimen and designed a randomized controlled trial to explore its efficacy and safety in the treatment of pain after VATS.

## Methods

### Study design and setting

This study will use a single-centre, parallel group, randomized controlled trial to explore the efficacy and safety of acupotomy combined with PCA for the treatment of pain after VATS. The study will be carried out at Fujian Provincial Hospital and has been registered with the Chinese Clinical Trial Registry (ChiCTR1900027191).

**Fig. 1:**
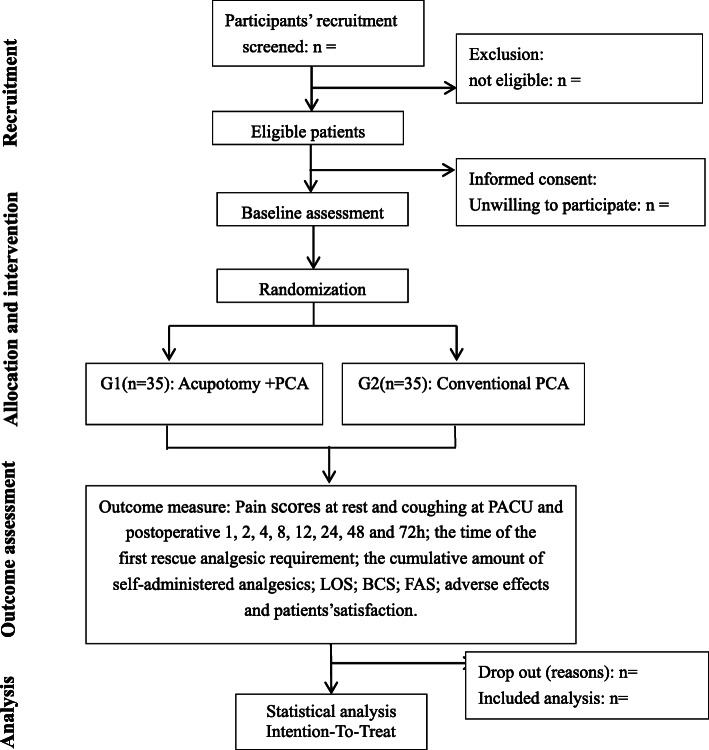
Flow diagram of the study design

After eligibility screening and written informed consent provision, eligible participants after thoracoscopic surgery will be randomly distributed into two postoperative analgesia groups: G1, the acupotomy combined with PCA group; and G2, the conventional PCA group. In addition to the abovementioned analgesic regimen, all subjects will receive conventional postoperative treatment, including health education and conventional medical therapy. This protocol design adheres to the Standard Protocol Items: Recommendations for Interventional Trials (SPIRIT) Checklist (Additional file 1). The flow diagram for this trial is presented in Fig. 1.

### Sample size

Based on the results of our preliminary experiments, the visual analogue scale (VAS) scores (mean difference ± SD) at rest for postoperative day 1 were 3.47 ± 1.39 in the conventional PCA group and 2.34 ± 1.21 in the acupotomy combined with PCA group. We input these data into the G-power 3.1.9.2 software (developed by the University of Düsseldorf, Germany) to calculate the sample size. We obtained a calculated effect size of 0.867 for this outcome after the intervention. A sample size of 70 participants is required to sufficiently detect a target effect size (0.8) with a type 1 error of 5% (*α* = 0.05). Therefore, 70 participants will be recruited, with 35 participants in each group.

### Participants and eligibility criteria

Competent doctors will strictly screen qualified subjects according to inclusion and exclusion criteria.

Seventy patients with lung cancer, an American Society of Anesthesiologists physical status I–II, an age of 30–70 years, and a VAS score ≥ 7 who are selected for VATS lobectomy will be recruited for this clinical study. They will also be required to be able to communicate well and understand how to score their pain level. All eligible participants who meet the study inclusion criteria will be identified through the Division of Thoracic Surgery. The study exclusion criteria are coagulation disorders; neuropathy; infections at the site of acupotomy; obesity (body mass index, BMI > 30 kg/m^2^); clinically significant neurological, cardiovascular, renal, and hepatic diseases; an inability to remove the tracheal catheter or to correctly use the PCA pump after surgery; psychiatric illnesses that would interfere with the perception and assessment of pain; and pain-killer use within a week before surgery.

We will have dedicated researchers to screen existing institutional registry records and databases and to work closely with the thoracic surgeons to identify qualified participants. Once eligibility is established, the research staff will contact eligible participants or their families and explain the purpose and significance of this study, provide full explanations to eliminate participants’ doubts, and ascertain interest in enrolment. If a participant agrees to enrol, informed consent will be obtained. Participants will undergo baseline assessments following informed consent provision. A CONSORT diagram of participant recruitment is shown in Table 1.

**Table 1:** Trial process chart

Items	Before enrollment -2-1 (weeks)	PACU	Postoperative 1 h	Postoperative 2 h	Postoperative 4 h	Postoperative 8 h	Postoperative 12 h	Postoperative 24 h	Postoperative 48 h	Postoperative 72 h
Inclusion criteria	×									
Exclusion criteria	×									
Informed consent	×									
Randomization and allocation	×									
**Pain scores** (VAS)		×	×	×	×	×	×	×	×	×
Postoperative requirements for rescue analgesia		×	×	×	×	×	×	×	×	×
LOS		×	×	×	×	×	×	×	×	×
BCS		×	×	×	×	×	×	×	×	×
FAS		×	×	×	×	×	×	×	×	×
Cumulative amount of self-administered analgesics								×	×	×
Adverse events		×	×	×	×	×	×	×	×	×
Patients’ satisfaction								×	×	×

### Randomization and blinding

In this study, we will use SAS (SAS 9.2) statistical software to create a randomization sequence generated by an independent statistician. The randomization sequence will assign patients on the basis of a 1:1 ratio to either the intervention group (acupotomy combined with PCA group) or the control group (conventional PCA group). The allocation sequence (containing random numbers and allocation and intervention information) will be concealed from the researchers who are responsible for enrolling and assessing participants in sequentially numbered, opaque, sealed, and stapled envelopes. The project manager will evaluate the baseline information of eligible participants and then inform them of the group to which they are assigned. Study personnel involved in recruitment, screening, data collection, and data entry will be blinded to group assignments. Due to the nature of the intervention, blinding the participants is impossible.

### Study intervention

#### Interventions, anaesthesia, and patient-controlled analgesia

All patients will be expected to avoid preoperative use of pain medication. After entering the operating room, clinicians will regularly monitor the patients’ blood pressure, electrocardiography, pulse oxygen saturation (SpO_2_), and end-tidal carbon dioxide tension (PETCO_2_).

Then, patients will receive general anaesthesia under a standard protocol. General anaesthesia will be induced with 0.05 mg/kg of midazolam, 2–4 μg/kg of fentanyl, and 2 mg/kg of propofol intravenously. Tracheal intubation will be facilitated with rocuronium bromide 0.6 mg/kg. If necessary, we will repeat intubation. We will use 1–2% sevoflurane to maintain anaesthesia. During the surgery, patients will intravenously receive propofol and remifentanil at doses of 2–2.5 μg/kg and 3–4 μg/kg, respectively. In addition, we will continuously administer cisatracurium besylate to maintain muscle relaxation. The oxygen concentration will be adjusted according to the arterial blood gas value of the partial arterial blood oxygen concentration value. PETCO_2_ will be controlled between 35 and 40 mmHg. A left-sided double-lumen endobronchial tube will be inserted under the premise that the tube size matches the left mainstem bronchial diameter. We will confirm the correct tube position with the help of a stethoscope and flexible fibreoptic bronchoscopy. At 30 min before the end of the operation, we will give the patients 5–10 mg of dezocine intravenously. Patients in the conventional PCA group will be immediately connected to an intravenous self-control analgesia pump containing 2 μg/ml of sufentanil and 8 mg of ondansetron diluted in 100 ml of 0.9% saline after surgery. The initial loading dose will be 2 ml, the background dose will be 2 ml/h, the single PCA dose will be 0.5 ml, and the locking time will be 15 min. Patients will not be treated with other medical analgesics. If special requirements emerge, the study will record the specific medication time, the drug name, each dose, the total dosage, and other information in detail. Patients in the acupotomy combined with PCA group will be treated with acupotomy before PCA treatment.

#### Acupotomy treatment

Senior traditional Chinese medicine (TCM) doctors with nearly 30 years of clinical experience in acupuncture and 5 years of acupotomology experience will perform all acupotomy procedures. The doctors will not be involved in evaluating the effects of treatment. A flat-head screw-driver-shaped stainless-steel disposable acupotomy needle (0.6 mm in diameter and 50 mm in length, Lejiu Acupotomy Company, China) will be used. Acupotomy will be performed in the lateral position. An iodine antiseptic will be used to sterilize the area for acupotomy. We will not anaesthetize the skin. The needle will be inserted at a position 0.5–1.5 in. away from the spinous process at the T4–T7 level on the painful side of the chest, 50–60 mm under the skin and in a direction parallel to the muscle fibres. The practitioner will stop advancing the acupotomy needle when resistance at the needle point is felt. The practitioner will move the needle point around in different directions to stimulate the soft tissue 3–5 times until the tenderness disappears. Then, the needle will be pulled out, and gauze will be applied to the site to prevent bleeding.

### Outcome measures

When patients are transferred to the PACU, vital signs (heart rate, noninvasive blood pressure, respiratory rate, and SpO_2_) will be monitored and recorded every 5 min for at least 30 min. When the patients’ vital signs are stable and they are able to communicate easily, a series of clinical-scale evaluations and analgesia will be evaluated and recorded. The primary outcome measure of this study is the postoperative pain intensity score. Pain scores (VAS) in the PACU and postoperatively at 1, 2, 4, 8, 12, 24, 48, and 72 h at rest and coughing in each group will be assessed. The VAS ranged from 0 = no pain to 10 = unbearable pain. The mean value and standard deviation will be used for descriptive analysis. The primary outcomes of this study were the resting and dynamic pain scores, which were recorded using a VAS. The secondary outcome measures are postoperative requirements for rescue analgesia, the cumulative amount of self-administered analgesics, the level of sedation (LOS), the Bruggemann comfort scale (BCS), and the functional activity score (FAS) concerning adverse effects and patient satisfaction.

The time to the first rescue analgesic use and the cumulative amount of self-administered analgesics in the first 48 and 72 h will be recorded. The BCS, LOS, and FAS will be recorded at 1, 4, 8, 16, 24, 48, and 72 h after surgery. The LOS is recorded on a 5-point scale, with 0 indicating fully awake, 1 indicating drowsy/closed eyes, 2 indicating asleep/easily aroused with light tactile stimulation or a simple verbal command, 3 indicating asleep/arousable only by strong physical stimulation, and 4 indicating unarousable. The BCS is scored as 0, persistent pain; 1, severe pain while deep breathing or coughing; 2, mild pain while deep breathing or coughing; 3, painless while deep breathing; and 4, painless while coughing. The FAS is scored as A, not restricted; B, mild to moderately restricted; and C, severely restricted. Postoperative adverse effects such as nausea, vomiting, hypotension, hypoxemia, cardiac arrhythmia, and the complications from the drugs and technique will be recorded and treated.

Patient satisfaction will be assessed verbally at 24, 48, and 72 h after surgery using a 5-point Likert scale (5 = completely satisfied, 4 = quite satisfied, 3 = slightly dissatisfied, 2 = dissatisfied, 1 = very dissatisfied). The observer who records the postoperative data will also be blinded to the group assignments.

### Reporting and treating adverse events

During the course of the study, researchers will record any AEs and then comprehensively evaluate the correlations between these AEs and the clinical intervention. To our knowledge, acupotomy may cause discomfort or bruising at the sites of needle insertion, nausea, or faintness after each treatment. No other serious adverse reactions caused by acupotomy have been reported to date. No harm or compensation is anticipated for trial participation. Meanwhile, if any AE occurs, the investigator will take proper measures to ensure the safety of participants; all details will be written down carefully.

### Data management and monitoring

The experimental data will be carefully entered into an electronic case report form (CRF). To guarantee the data quality, we will appoint a dependent research assistant (RA) to be responsible for the quality control during the data collection process. Participant identification and privacy information will be deleted from all study documents to protect confidentiality. All outcomes will be manually double-checked, and a second independent data entry will be conducted to promote data quality. After data storage, only researchers directly involved in the data analysis will be permitted to access to the final trial dataset. During the study, an independent data and safety monitoring board (DSMB) of the scientific research department of Fujian Provincial Hospital will be responsible for monitoring the safety, progress, study integrity, and design aspects of the trial. Drop-outs and withdrawals from the trial will be recorded throughout the intervention, and the data will be collected in the CRF and used for the final statistical analysis. No conflict of interest with the sponsors or researchers exists.

### Statistical analysis

The statistical analysis will be carried out using SAS (SAS 9.2) statistical software. All allocated subjects with available data will be analysed, i.e. on the basis of the intention-to-treat principle. The continuous variables will be presented as the means ± SDs (standard deviations) or the medians (25th to 75th centiles) and compared using the two-tailed Mann-Whitney test or Student’s *t* test, as appropriate. Categorical variables will be presented as numbers and percentages and analysed by Fisher’s exact test or the chi-squared test. Repeated measurements will be analysed using two-way ANOVA, and Tukey’s test will be used for post hoc testing. All statistical tests will be performed with a bilateral test, and *P* ≤ 0.05 will be considered as statistically significant.

## Discussion

This prospective randomized control study aims to evaluate the effects of acupotomy combined with PCA (compared to conventional PCA) on postoperative pain, functional recovery, postoperative adverse effects, and patient satisfaction among patients undergoing VATS with poorly controlled postoperative pain. Despite the application of minimally invasive techniques, improved administration of analgesic drugs, and combined use of analgesic drugs, the postoperative pain of some patients after thoracoscopic surgery is not well controlled [22].

As a concept for a multimodal perioperative analgesic regimen, the options for postoperative analgesia must be effective with minimal side effects and aim to decrease the potentially harmful consequences of thoracic surgery on immediate and long-term patient well-being [23, 24]. Moreover, as improving patients’ recovery after surgery and reducing patients’ hospital stay are key aims of fast-track surgery, other pain-relieving strategies must be considered to complement the analgesics currently used [25–27].

PCA is a common analgesic method after thoracoscopic surgery in clinical practice [1, 28], and acupotomy is a traditional Chinese acupuncture treatment focusing on the pain treatment of clinical diseases [29, 30]. The combination of acupotomy and conventional PCA can provide new insights into complicated postoperative pain management and functional rehabilitation of patients after thoracoscopy.

In this study, we will use a combination of patient-reported subjective evaluation scales and objective statistical evaluation indicators of the effect of analgesic use to verify the effect of acupotomy combined with PCA on pain after thoracoscopy, thus increasing the reliability of the research results. In addition, early rehabilitation of postoperative limb function is also an important part of evaluating the effect of surgical treatment. In this study, the FAS scale will be used to evaluate the recovery of upper limb motor function in patients after thoracoscopic surgery. At the same time, we will also focus on evaluating adverse reactions and patient satisfaction. To the best of our knowledge, this will be the first randomized controlled trial to study the effect of a combination of acupotomy and PCA on the management of postoperative pain and functional recovery in this population.

The major limitation of this protocol is its non-double-blind design. Nevertheless, we have taken some remedial measures to further ensure the quality of the research. For instance, patients will be allocated using a computerized randomization schedule to receive either of two techniques, which may control for selection bias. Moreover, the outcome assessors and statistical analysts will be blind to the intervention, which may control for report bias. Another limitation of this study is that no blank control group was established to exclude the placebo effect. We will consider this issue in the next more rigorous experimental design. This study also lacks long-term follow-up observations and assessments. The 3-day continuous observation period reflects the real-world clinical practice of the observation of acute analgesia after thoracoscopy and is sufficient to confirm the clinical efficacy and safety of acupotomy combined with PCA in the short-term treatment of pain after thoracoscopy.

In conclusion, our results may offer an innovative strategy for improving postoperative pain control that could have a substantial impact on postoperative care and quality of life for patients undergoing VATS.

### Trial status

Protocol number: version 2.0-2019.12.01. Recruiting start date: December 5, 2019. Expected study completion date: December 2021. In case of any changes to the protocol, we will notify the relevant parties (investigators, participants, etc.) through e-mail or letter.

## Supplementary Information

**Additional file 1.** SPIRIT checklist.

**Additional file 2.** Acupotomy operation

## Data Availability

The datasets used and/or analysed during the current study will be available from the corresponding author on reasonable request following the completion of the trial.

## Abbreviations

PCA: Patient-controlled analgesia
VATS: Video-assisted thoracic surgery
VAS: Visual analogue scale
PACU: Postanaesthesia care unit
LOS: The level of sedation
BCS: Bruggemann comfort scale
NSAID: Systemic nonsteroidal anti-inflammatory drug
FAS: Functional activity score
ERAS: Enhanced Recovery After Surgery
AEs: Adverse events
PETCO2: End-tidal carbon dioxide tension
SpO2: Pulse oxygen saturation

## References

[1] Jin J, Min S, Chen Q, Zhang D (2019). Patient-controlled intravenous analgesia with tramadol and lornoxicam after thoracotomy: a comparison with patient-controlled epidural analgesia. Medicine (Baltimore).

[2] Dai F, Meng S, Mei L, Guan C, Ma Z (2016). Single-port video-assisted thoracic surgery in the treatment of non-small cell lung cancer: a propensity-matched comparative analysis. J Thorac Dis.

[3] Zhu Y, Liang M, Wu W, Zheng J, Zheng W, Guo Z, Zheng B, Xu G, Chen C (2015). Preliminary results of single-port versus triple-port complete thoracoscopic lobectomy for non-small cell lung cancer. Ann Transl Med.

[4] Maguire MF, Latter JA, Mahajan R, Beggs FD, Duffy JP (2006). A study exploring the role of intercostal nerve damage in chronic pain after thoracic surgery. Eur J Cardiothorac Surg.

[5] Xu J , Yang X , Hu X , et al. Multiple-level Thoracic Paravertebral Block Using Ropivacaine with/without Dexmedetomidine in Video-Assisted Thoracoscopic Surgery[J]. J Cardiothorac Vasc Anesth. 2017;32(1). 10.1053/j.jvca.2017.06.023. 10.1053/j.jvca.2017.06.023 29191649

[6] Lovich-Sapola J, Smith CE, Brandt CP (2015). Postoperative pain control. Surg Clin North Am.

[7] Hiro K, Sugiyama T, Kurata M, Oi Y, Okuda M (2016). Postoperative analgesia for video-assisted thoracoscopic surgery--continuous intravenous infusion of fentanyl combined with intercostal nerve block v.s. continuous epidural analgesia. Masui.

[8] Yie JC, Yang JT, Wu CY, Sun WZ, Cheng YJ (2012). Patient-controlled analgesia (PCA) following video-assisted thoracoscopic lobectomy: comparison of epidural PCA and intravenous PCA. Acta Anaesthesiol Taiwanica.

[9] Ong CK, Lirk P, Seymour RA, Jenkins BJ (2005). The efficacy of preemptive analgesia for acute postoperative pain management: a meta-analysis. Anesth Analg.

[10] Fibla JJ, Molins L, Mier JM, Sierra A, Carranza D, Vidal G (2011). The efficacy of paravertebral block using a catheter technique for postoperative analgesia in thoracoscopic surgery: a randomized trial. Eur J Cardiothorac Surg.

[11] Chen N, Qiao Q, Chen R, Xu Q, Zhang Y, Tian Y (2019). The effect of ultrasound-guided intercostal nerve block, single-injection erector spinae plane block and multiple-injection paravertebral block on postoperative analgesia in thoracoscopic surgery: a randomized, double-blinded, clinical trial. J Clin Anesth.

[12] Unic-Stojanovic D, Babic S, Jovic M (2012). Benefits, risks and complications of perioperative use of epidural anesthesia. Med Arch.

[13] Freise H, Van Aken HK (2011). Risks and benefits of thoracic epidural anaesthesia. Br J Anaesth.

[14] Ljungqvist O, Scott M, Fearon KC (2017). Enhanced recovery after surgery: a review. JAMA Surg.

[15] Beverly A, Kaye AD, Ljungqvist O, Urman RD (2017). Essential elements of multimodal analgesia in enhanced recovery after surgery (ERAS) guidelines. Anesthesiol Clin.

[16] Bertolaccini L, Brunelli A (2019). Devising the guidelines: the techniques of uniportal video-assisted thoracic surgery-postoperative management and enhanced recovery after surgery. J Thorac Dis.

[17] Guthrie MP, Xhaja A, Beck AW. Improving lower extremity bypass patient outcomes: enhanced recovery after surgery implementation project. J Nurs Care Qual. 2019, publish ahead of print(1):1. 10.1097/NCQ.0000000000000431. 10.1097/NCQ.0000000000000431 31464845

[18] Piccioni F, Segat M, Falini S, Umari M, Putina O, Cavaliere L, Ragazzi R, Massullo D, Taurchini M, Del NC, Droghetti A (2018). Enhanced recovery pathways in thoracic surgery from Italian VATS Group: perioperative analgesia protocols. J Thorac Dis.

[19] Li S, Shen T, Liang Y, Zhang Y, Bai B (2015). Effects of miniscalpel-needle release on chronic neck pain: a retrospective analysis with 12-month follow-up. PLoS One.

[20] Jang EH, Kim SY, Kim HS, Kim SC (2009). Acupotomy and venesection in upper limb lymphedema and peripheral neuropathy following breast cancer surgery. J Pharmacopunct.

[21] Park JY, Park JJ, Jeon S, Doo AR, Kim SN, Lee H, Chae Y, Maixner W, Lee H, Park HJ (2014). From peripheral to central: the role of ERK signaling pathway in acupuncture analgesia. J Pain.

[22] Bai Y, Sun K, Xing X, Zhang F, Sun N, Gao Y, Zhu L, Yao J, Fan J, Yan M (2019). Postoperative analgesic effect of hydromorphone in patients undergoing single-port video-assisted thoracoscopic surgery: a randomized controlled trial. J Pain Res.

[23] Wick EC, Grant MC, Wu CL (2017). Postoperative multimodal analgesia pain management with nonopioid analgesics and techniques: a review. JAMA Surg.

[24] Ren C, Zhang X, Liu Z, Li C, Zhang Z, Qi F (2015). Effect of intraoperative and postoperative infusion of dexmedetomidine on the quality of postoperative analgesia in highly nicotine-dependent patients after thoracic surgery: a CONSORT-prospective, randomized, controlled trial. Medicine (Baltimore).

[25] Holbek BL, Horsleben PR, Kehlet H, Hansen HJ (2016). Fast-track video-assisted thoracoscopic surgery: future challenges. Scand Cardiovasc J.

[26] Kehlet H, Wilmore DW (2008). Evidence-based surgical care and the evolution of fast-track surgery. Ann Surg.

[27] Xia Y, Chang S, Ye J, Xue J, Shu Y (2016). Application effect of fast track surgery for patients with lung cancer: a meta-analysis. Zhongguo Fei Ai Za Zhi.

[28] Zhou Y, Huang JX, Lu XH, Zhang YF, Zhang W (2015). Patient-controlled intravenous analgesia for non-small cell lung cancer patient after thoracotomy. J Cancer Res Ther.

[29] Liu F, Zhou F, Zhao M, Fang T, Chen M, Yan X (2017). Acupotomy therapy for chronic nonspecific neck pain: a systematic review and meta-analysis. Evid Based Complement Alternat Med.

[30] Kwon CY, Yoon SH, Lee B (2019). Clinical effectiveness and safety of acupotomy: an overview of systematic reviews. Complement Ther Clin Pract.

