# Supplementary figures and images for: Postoperative analgesic effect of acupotomy combined with patient-controlled analgesia in patients undergoing video-assisted thoracoscopic surgery: a study protocol for a randomized controlled trial
Source: Trials. 2020 Dec 4;21:998. doi: 10.1186/s13063-020-04926-7 (PMC7716496; doi:10.1186/s13063-020-04926-7)

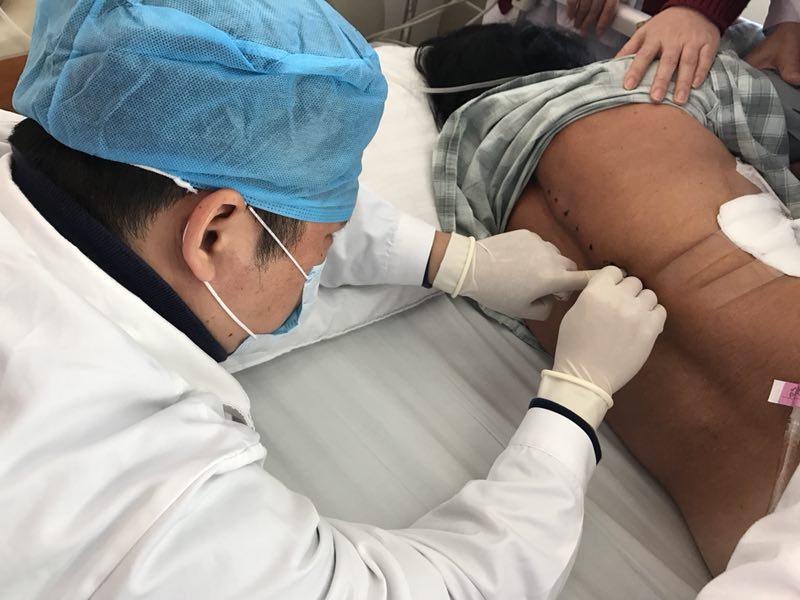

Supplement: Supplementary file 2 — Additional file 2. Acupotomy operation [file 13063_2020_4926_MOESM2_ESM.jpg]
